# A Natural History of Disease Framework for Improving the Prevention, Management, and Research on Post-viral Fatigue Syndrome and Other Forms of Myalgic Encephalomyelitis/Chronic Fatigue Syndrome

**DOI:** 10.3389/fmed.2021.688159

**Published:** 2022-01-28

**Authors:** Shennae O'Boyle, Luis Nacul, Flavio E. Nacul, Kathleen Mudie, Caroline C. Kingdon, Jacqueline M. Cliff, Taane G. Clark, Hazel M. Dockrell, Eliana M. Lacerda

**Affiliations:** ^1^Faculty of Infectious and Tropical Diseases, London School of Hygiene & Tropical Medicine, London, United Kingdom; ^2^UK Health Security Agency, London, United Kingdom; ^3^B.C. Women's Hospital and Health Centre, Vancouver, BC, Canada; ^4^Pro-cardiaco Hospital and Federal University of Rio de Janeiro, Rio de Janeiro, Brazil; ^5^Faculty of Epidemiology and Population Health, London School of Hygiene & Tropical Medicine, London, United Kingdom

**Keywords:** myalgic encephalomyelitis/chronic fatigue syndrome, chronic fatigue syndrome, ME/CFS, post-viral fatigue syndrome, chronic illness, management, research

## Abstract

We propose a framework for the treatment, rehabilitation, and research into Myalgic Encephalomyelitis/Chronic Fatigue Syndrome (ME/CFS) using a natural history of disease approach to outline the distinct disease stages, with an emphasis on cases following infection to provide insights into prevention. Moving away from the method of subtyping patients based on the various phenotypic presentations and instead reframing along the lines of disease progression could help with defining the distinct stages of disease, each of which would benefit from large prospective cohort studies to accurately describe the pathological mechanisms taking place therein. With a better understanding of these mechanisms, management and research can be tailored specifically for each disease stage. Pre-disease and early disease stages call for management strategies that may decrease the risk of long-term morbidity, by focusing on avoidance of further insults, adequate rest to enable recovery, and pacing of activities. Later disease stages require a more holistic and tailored management approach, with treatment—as this becomes available—targeting the alleviation of symptoms and multi-systemic dysfunction. More stringent and standardised use of case definitions in research is critical to improve generalisability of results and to create the strong evidence-based policies for management that are currently lacking in ME/CFS.

## Introduction

Myalgic Encephalomyelitis/Chronic Fatigue Syndrome (ME/CFS) is a complex disease of unknown aetiology with no diagnostic test or biomarker to enable accurate and timely identification of cases (1). Diagnosis is often delayed by years, due to factors including: (i) the marked heterogeneity of the disease, (ii) the extensive clinical investigations necessary to exclude alternative diagnoses, and (iii) the numerous case definitions available, which differ significantly (1). Without a specific diagnostic test, identification of ME/CFS cases largely relies on detailed clinical history and examination, together with the patients' description of current and past symptoms. Many cases are triggered by an infection (also known as post-infectious ME/CFS) and there is a growing body of literature that reports clusters of ME/CFS cases following viral infections, although it is often not possible to confirm the triggering infection (2–6). The UK ME/CFS Biobank (UKMEB) has a cohort of 306 consenting participants diagnosed with ME/CFS, of whom 68% (when recalling disease onset) reported having a viral infection prior to the start of symptoms; 35% of these participants reported that their viral infections were confirmed by laboratory tests.

The difficulties in diagnosing patients have a knock-on effect for both clinical management and research: the amalgamation of people with ME/CFS and those with chronic fatigue due to other causes (e.g., conditions such as diabetes or anaemia that are not adequately controlled with medication) contributes to a lack of specificity (7, 8), while the numerous case definitions and inconsistent subtyping exacerbate heterogeneity and non-generalisability of study results (1). In addition, retrospective study designs including participants with established disease inevitably neglect the crucial period of pre- or early disease (where disease is developing and progressing), and instead rely on patient recall in order to gather information for that period. Disease duration is critical for diagnosis; most current definitions require that symptoms be present for at least 6 months for a formal ME/CFS diagnosis to be considered (9, 10), and this, together with a lack of biomarkers, makes it nearly impossible to identify those people predisposed to, or in the early stages, of disease progression. Larger prospective cohort studies following acute infections are required to accurately describe disease progression, and to identify specific markers for each disease stage (11–13).

With this conceptual paper, we intend to outline the stages of this disease for the optimisation of treatment, rehabilitation, and research into ME/CFS by considering specific preventative measures, improving generalisability of results, and creating the strong evidence-based policies for management that are currently lacking in ME/CFS. This paper sets out how research and clinical management could be targeted to specific disease phases, with a focus on prevention and rehabilitation, to improve patient outcomes.

### The Natural History of ME/CFS

In an earlier paper, we conceptualised the progression of ME/CFS using a natural history of disease framework (further summarised below) (14); a concept familiar to many other chronic diseases. By considering each distinct stage along a chronological development timeline, we can move away from the multitudes of ways patients have previously been characterised including, but not limited to, the following: symptom presentation (15); co-morbidities (16); genetic traits (17, 18); metabolomics (19); and disease duration (16, 20), enabling an initial alignment of disease stage, clinical phenotype and potential pathophysiological mechanisms (14). While ME/CFS aetiology and its pathophysiological mechanisms remain elusive at some of the stages, this proposed framing draws attention to the less defined pre-morbid phases, the understanding of which may be the key to identifying the early causes of ME/CFS, and where early intervention may be effective. The proposed stages are as follows:

### Predisposition and Triggering of Disease (Onset)

This is the period before disease is initiated in the individual. Without a full understanding of disease aetiology, it remains unclear which individuals are predisposed, but there are certain well-accepted patterns including: gender- and age-specific factors (21–23); acute infection triggers, either sporadic or as part of outbreaks (24–28); and genetic heritability (29, 30). There are a number of other factors reported as triggers including stress, environmental causes and trauma (31–34). Most commonly, ME/CFS develops following an acute viral episode (of which various aetiologies have been noted) (3); other patients report a slower, more insidious onset with no obvious initiating factor (35). At these early stages, disease presentation is non-specific or related to the “triggering” insult. Current reports of chronic symptoms similar to those of ME/CFS have been described by people infected with SARS-CoV-2 (36–38).

### Prodromal Period (0–4 Months)

A lack of research makes it difficult to substantiate exactly what happens during the prodromal (and early disease) stage, although the mechanisms involved in producing the first symptoms of ME/CFS likely result from the bi-directional interaction between the immune and the central nervous systems (CNS), pro-inflammatory cytokines and other mediators disrupt CNS function which, in turn, releases neurotransmitters and hormones affecting immune function (39–41). Consequently, the hypothalamic-pituitary-adrenal (HPA) axis and the autonomic nervous system (ANS) are affected, interrupting the normal homeostatic processes in the body (42–45).

### Early Disease (4–24 Months)

This stage represents the continued and increasing dysregulation initiated in the prodromal period, where physiological and homeostatic processes are unable to return to previous levels of equilibrium and instead settle into a new “aberrant” homeostatic rhythm: an alternative state of functioning at a less optimum level (46). Symptoms such as fatigue can be largely explained by local and systemic effects of cytokines (47) or toxins and systemic dysfunction (48–52). There is a shift towards conservation of energy for essential processes, and physiological responses and symptoms are modulated by the increased production of anti-inflammatory mediators to balance out the pro-inflammatory stimuli.

### Established ME/CFS (2 Years and Beyond)

The initial over-production of pro-inflammatory and neurotoxic factors and ongoing immune and CNS dysfunction leads to a prolonged state of low-grade neurological and systemic inflammation (31, 35, 39, 53–61). With time, there is a shift from a higher to a less active pro-inflammatory state (62), with possible changes to symptom severity. However, individuals may move between phases either upwards (i.e., towards homeostasis and better health status) or downwards (i.e., towards “aberrant homeostasis” and disease deterioration).

By considering the distinct disease stages in ME/CFS, management of symptoms will inevitably be improved, leading to an increased likelihood of recovery. This is because the distinct stage-specific mechanisms underpinning pathology, require differential measures that may help to restore normal functioning. Currently, the lack of research at the prodromal and early disease phases (compounded by the discrepant use of case definitions and subtyping described above) means treatment is limited to managing symptoms rather than tackling their cause. In the absence of recognised treatments, the best approach for people with ME/CFS would be preventative measures combined with symptoms management at each phase of disease.

## Methodological Approach

**Figure 1:**
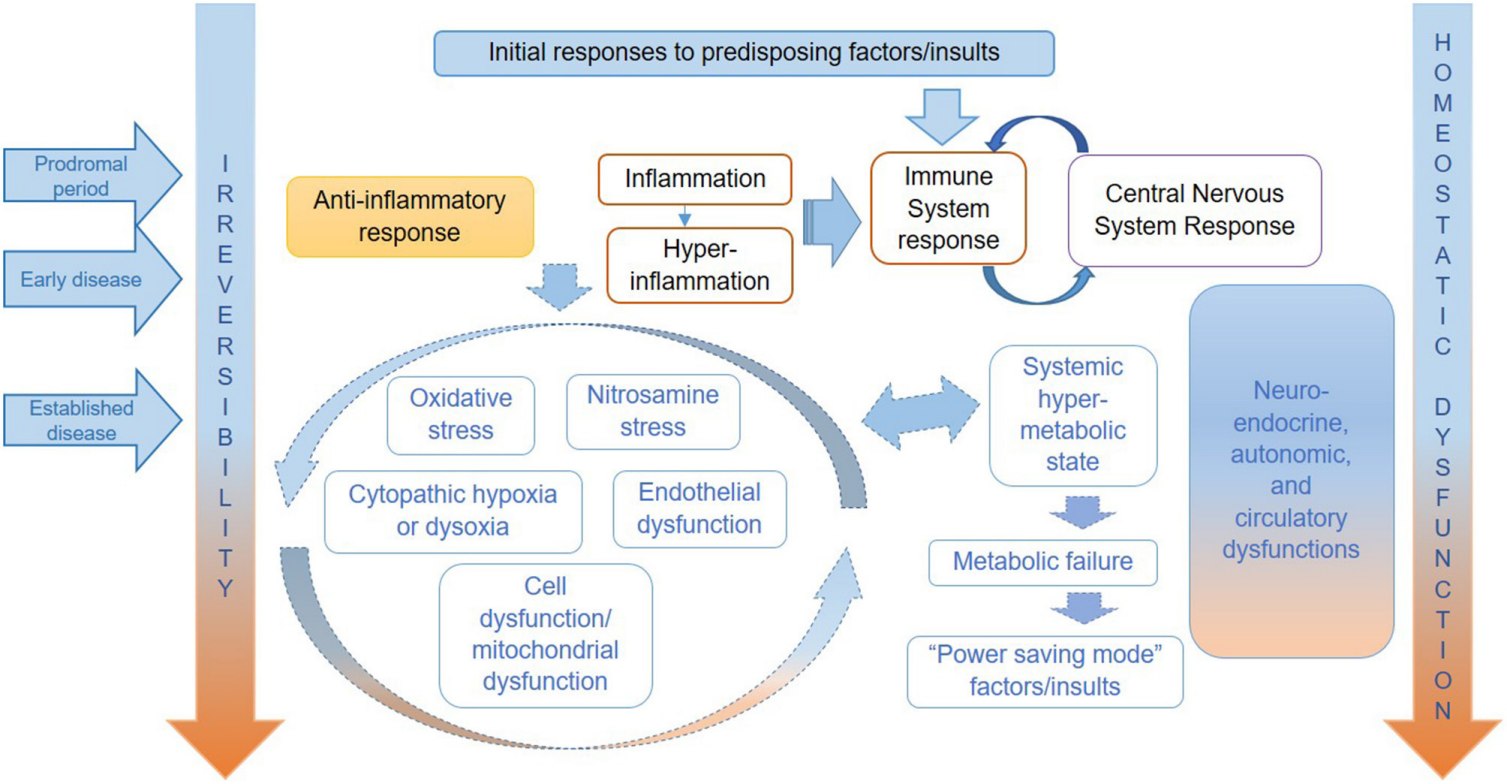
Hypothesised key pathophysiological mechanisms for ME/CFS.

In this theoretical paper, we have applied the natural history of ME/CFS framework to consider preventative measures, management and treatment of symptoms, and research targeted to specific stages of disease. We argue that the proposed framework will help to target the public health, clinical, and research efforts in ME/CFS in more effective ways, recognising that it will likely be improved by future research findings. Table 1, adapted from our previous conceptual paper on a proposed natural history of this disease (14), shows the putative stages of ME/CFS—from predisposition to established disease, which are correlated to clinical phenotypes (defined by symptoms)—and the possible prevention and disease management strategies. Figure 1, copied from Nacul et al., attempts to illustrates the key pathophysiological mechanisms operating in each stage of ME/CFS, based on current literature (14).

**Table 1:** Characterisation of ME/CFS progression on time, according to distinct stages from pre- to established, clinical phenotype, and levels of prevention.

**Timing**	**No disease**	**Onset**	**0–4 months**	4–24 months^*^	**2 years+ ^†^**
Stage	Predisposition	Trigger and pre-illness	Prodromal period	Early disease	Established disease
Clinical phenotype	No symptoms	Non-specific or related to triggering “insult”	Fatigue-complex symptoms^‡^	Fatigue-complex symptoms with variable severity and progress	Mild, moderate, or severe and complicated disease
Prevention level	Primary prevention	Treatment of “insult” and primary prevention	Symptoms management and secondary prevention	Disease management and secondary prevention	Disease management and tertiary prevention

*Table adapted from Nacul et al. (14)*.

* *3–6 months is commonly referred as the minimum period of symptoms before diagnosis is made in children and adults, respectively (35)*.

† *2 years has been used as a cut off to distinguish between short- and long-term duration of disease (63, 64), but its use as defining established disease is variable and depends on a range of factors, including individual response to early disease*.

‡ *Fatigue-complex symptoms: initially predominantly neuro-immune (prior to early disease) and progress to variable systemic symptoms in the established disease phase*.

## Levels of Prevention, Management, and Research Based on The Distinct Stages of ME/CFS

### Predisposition and Triggering of Disease (Onset)

#### Primary Prevention

While risk factors specific to ME/CFS remain ambiguous it is difficult to conceptualise, or even put into practise, evidence-based primary prevention strategies. It is reasonable to assume that, in the face of acute infections or other insults, individuals should avoid exposure to further stresses and prioritise periods of relative rest along with pacing activities (as appropriate) in order to facilitate recovery from acute illness. This requires support from employers, teachers (see section below for the role of “presenteeism”), and healthcare professionals. For cases that might be triggered by environmental contaminants (such as chemicals), environmental protection policies and regulation will play an important role (65).

#### Management

At this predisposition stage, potential management measures are quite limited, and would overlap with the primary preventative measures, if the triggers are identified as infection-related as described above.

#### Research

Any change in practise will result from a better understanding of risk factors specific to ME/CFS and from research with the design of a disease-specific strategy for ME/CFS prevention, which is currently lacking. This strategy should be considered within the context of wider determinants of health (66), using a model that applies to the prevention of chronic diseases in general and considers potential predisposing factors, including genetics (67–70). Knowledge of risk factors for ME/CFS, currently scarce, are essential for primary prevention and we therefore recommend research approaches used in the study of other chronic diseases to gain new insights into familial and individual risks, including genetic, environmental, and life-style factors (2). Examples of proxy models that could be used to further our understanding of risk factors and the immune response in ME/CFS include interferon-alpha (IFN-α) treatment for hepatitis C that was suggested to trigger chronic fatigue (71), cohort studies that follow fatigue after infection with Epstein-Barr Virus (EBV) (11), and more recently, SARS-CoV-2 (72).

In the first example, considering IFN-α as a “trigger” allows for observation and tracking of the disease profile prior to, during, and after the presence of the insult, and following cohorts of patients from an early stage further allows the identification of possible risk factors and biomarkers. While appreciating that distinct mechanisms may be at play in ME/CFS, it is reasonable to consider similar proxy models to seek better understanding of immune profiles and response to insults in other fatiguing diseases. Following disease progression in people infected with SARS-CoV-2 in the current pandemic using well-designed studies may answer some questions about the potential chronic responses to viral infections, including whether long-COVID can be defined as a similar or distinct disease (73). Furthermore, such studies would provide an opportunity for improved real-time characterisation of the natural history of disease.

### Prodromal Period and Early Disease Phases

#### Secondary Prevention

Secondary prevention refers to early detection of a disease and to early intervention, with the aim of reducing morbidity and disability (74). In the case of ME/CFS, early diagnosis would have an impact on disease management, even in the absence of any specific treatment. In order to facilitate this, a provisional diagnosis of ME/CFS should be considered earlier (e.g., after 2 months of symptoms), with efforts made to regularly monitor patients until the disease can be confirmed at 4 to 6 months, after which regular reviews should continue (35).

Approaches towards early diagnosis require a major shift in perspective from both healthcare professionals and patients. For example, at the stage when a diagnosis of ME/CFS (or similar post-viral fatigue syndromes) is a possibility, we recommend a reduction in allostatic load including in activity levels (75–77), the avoidance of further stressors, and the treatment of the infection or triggering factor(s), when possible. Techniques such as pacing require substantial behaviour change in the patient firstly, to identify their energy threshold and, secondly, to adjust activity accordingly to avoid symptom exacerbation (i.e., keeping within the “energy envelope”) (78, 79). Additionally, any specific therapies should be aimed at the correction of immune, CNS, and other dysfunction, alongside prevention of complications.

Strong support from educational institutions and workplaces is critical at this stage of the disease process. Accommodating the need for adequate recovery time away from, or with reduced time at, work or studies (80) is paramount, particularly with growing evidence of the higher costs of presenteeism (i.e., being physically at work, even if ill) compared to absenteeism, and the adverse effects on an individual's own health and productivity when turning up to work ill (81). Providing such support might require society as a whole to recognise the importance of the needs of the individual for adequate recovery time following acute illness and of taking the pressure off individuals to be productive or present in their workplaces or classrooms at such times. This would avoid or minimise negative impacts on their health in the short- and long- term, as increased or persistent exertion (whether it is physical or mental) may result in the worsening of symptoms and a delay in recovery (35).

#### Management

Until there is conclusive evidence of specific pathophysiological mechanisms and effective treatments in ME/CFS, disease management at this stage should focus on reducing the severity of symptoms. Effective treatment plans rely on a strong health professional-patient relationship with regular follow-ups and a cautious degree of trial and error in treatment approaches (including starting any medications at low doses to monitor any sensitivities) (82, 83); such management should be informed by symptom characteristics and by awareness of changing symptoms which may reflect drug sensitivities or progression to later phase of disease. Although, not recommended specifically for the treatment of ME/CFS, a number of drugs have been shown in clinical practise to be helpful in some individuals for symptom management of pain (e.g., Low-dose naltrexone, Pregabalin, Gabapentin), orthostatic intolerance (e.g., Fludrocortisone, Midodrine), allergic/inflammatory reactions (e.g., antihistamines, sodium cromoglicate) and sleep (e.g., trazodone, Melatonin, tricyclics) (83). Non-pharmacological and behavioural approaches can also be helpful to relieve exacerbation of symptoms: acupuncture to assist with pain; support stockings and fluid/salt intake for orthostatic symptoms; memory aids and lists to help with cognitive issues; avoidance of specific foods and/or environmental factors (such as light, noise, touch etc.) (35, 83–86).

Management should be multidisciplinary, based on ongoing dialogue and partnership between professionals and patients extending to carers and family, with the involvement of the educational, occupational, and social sectors as appropriate.

With large numbers of the global population exposed to a potential viral trigger during this current SARS-CoV-2 pandemic, it is reasonable to suppose that a significant number of people with long-COVID may have, or will develop, ME/CFS. It is estimated that 1 in 10 people are experiencing persistent symptoms for over 12 weeks (87), often despite a relatively “mild” acute illness and reports of previously healthy lifestyles (88, 89), and are consequently diagnosed with long-COVID, post-COVID fatigue, or long-COVID fatigue syndrome (LCFS) (72, 73, 90).

There is much overlap in LCFS and ME/CFS symptom presentation (72), but it remains unclear whether they are the same condition. In the absence of that evidence, it would seem prescient to manage symptoms of LCFS by encouraging the use of recognised rehabilitation techniques long used for the management of ME/CFS (such as pacing) to reduce the likelihood of progression to post-COVID ME/CFS (91); while avoiding harmful treatments, such as the long-contested graded exercise therapy (92). Online long-COVID support groups report narratives similar to those of people with ME/CFS including being “dismissed” by healthcare professionals and labelled with “anxiety” (93). As healthcare workers themselves are diagnosed with long-COVID (94), such reports are being taken more seriously and there is growing awareness of the need for better recognition and management of post-COVID fatigue that is helping to drive change in the healthcare perspective (94, 95).

#### Research

In relation to secondary prevention, we believe that research should focus on the pathophysiology of early disease and early interventions at sub-clinical, acute, and early disease stages, and should target those factors that facilitate and hamper recovery from acute disease. Again, this would require prospective follow up of relatively large cohorts of individuals from exposure to an insult [such as an acute infection (11)], and thereafter in order to look at any differences between those who develop prolonged, chronic fatigue including ME/CFS and those who present no fatigue or with fatigue for shorter periods.

Within the field of ME/CFS research, studies using small participant numbers and a variety of diagnostic criteria are commonplace and lead to non-replicable or non-comparable results. The ongoing study of risk factors and potential biomarkers [of which a number of candidates have been considered (96, 97), although remain unconfirmed] may further benefit from existing large datasets [e.g., GP electronic health record databases for research (https://www.pcrd.purdue.edu/)]; or from general [e.g., UK Biobank (https://www.ukbiobank.ac.uk/)], and/or disease-specific databases and biobanks [e.g., UKMEB (https://cureme.lshtm.ac.uk/) and the SolveME/CFS Initiative (https://solvecfs.org/)].

Bioresources such as the UKMEB, which uses a strict protocol for the recruitment of participants and the collection and storage of biosamples (98), would serve to improve the replicability of studies by minimising the variation in sampling by the use of shared diagnostic criteria, and to provide samples for validation studies. Other opportunities include the application of life course epidemiology methods (99) on existing disease cohorts (that have well-documented medical histories) for diagnostic confirmation of ME/CFS, using retrospective or prospective longitudinal designs. Approaches could target individual, environmental, or genetic factors. Further genomic association studies could look at the association of candidate genes with disease, based on hypotheses from the evolving understanding of disease mechanisms at the molecular level; genetic family studies and large genome wide association studies could also contribute to identifying new susceptibility mechanisms.

### Established Disease (More Than 2 Years)

#### Tertiary Prevention

Tertiary prevention refers to actions aimed at reducing the impact of long-term illness and resulting morbidity and disability (74), including through rehabilitative interventions. The absence of an evidence-based curative treatment should not detract from the main objective of supporting the individual and of managing symptoms and disability. A relatively new concept, quaternary prevention originally proposed by Jamoulle (100), has been conceptualised by Martins et al. (101) as “*the action taken to protect individuals (persons/patients) from medical interventions that are likely to cause more harm than good*”, with the aim being “*to reduce over-medicalisation and iatrogenic harm*”. It is important that health professionals share decision-making with patients around the use of personalised treatments because of the wide range of often non-evidence-based therapies used for ME/CFS; these include alternative health practises (102) and behaviour-based therapies (103). Decision-making should be well-informed and acknowledge the availability (or lack of) evidence for the potential benefits and/or risks of treatments, while also considering the individual and the service costs of such treatments.

All rehabilitation strategies must be based on the understanding of the pathophysiology of severe and complicated disease and disability at individual, service, and societal levels. Tertiary prevention may improve with increased research focused on this specific stage of disease.

#### Management

Ideally, management of established ME/CFS should centre on the restoration of a healthier homeostatic balance through specific treatments and avoidance of aggravating factors, but the lack of sufficient treatment evidence necessitates limiting such management to life-style changes [including advice on planning and executing activities within the individual's energy limit levels (79)], and the use of symptomatic medications, such as analgesics and sleep medications (10), and the avoidance of other causes of neuro-immune overload, as described previously (83, 86). As evidence grows, treatments targeting multi-systemic abnormalities (such as those resulting from dysfunctions in the immune, neuro-endocrine, autonomic, circulatory, and neuro-muscular systems) will be critical for disease management; examples could include immune-based treatments, and those targeting oxidative stress and metabolic abnormalities.

#### Research

For established disease, research efforts should target the understanding of mechanisms that perpetuate abnormalities and the better understanding of pathways to recovery, including specific treatments targeted at various system and molecular abnormalities. Longitudinal studies are critical to address temporal pathophysiological changes in order to guide therapeutic approaches at different disease stages, and to investigate short- and long-term complications, including co-morbidities and mortality. Younger patients with a shorter duration of disease have been found to present with different phenotypes, in relation to autonomic nervous system manifestations (16), for example, and are therefore more likely to require specific treatment for postural orthostatic tachycardia syndrome (POTS) or postural hypotension symptoms. Description and/or comparisons of specific subtypes through longitudinal studies would help to determine differences in phenotype and encourage a more tailored approach to treatment management. Research on perceptions and attitudes to prolonged illnesses, from the individual, family, educational, occupational, and wider societal points of view, would help to change the way ME/CFS is managed, which would, in turn, help with secondary prevention.

Over 15 years ago, Bell wrote “*we need to change the focus of our telescope from looking at large organs to looking at single cells*” as he considered the search for evidence in ME/CFS (104). As we focus on the cellular level of molecular and systems medicine and transfer knowledge acquired from other conditions (such as acute severe injury), we should get closer to finding the real explanations for the various subgroups in ME/CFS. The “omics” technologies (e.g., transcriptomics, metabolomics, proteomics and genomics, including pharmacogenomics) are becoming increasingly accessible. Meaningful and translatable research outputs based on relevant research questions are now possible, as long as strong methodological approaches are applied. These should cover research design and case selection, sampling and management of bio-specimens, and appropriate application of technology and interpretation of findings.

As disease understanding evolves, we will move closer to personalised health care and medicine, and more specific strategies for prevention and treatment will become possible (105). Examples of such strategies include the targeting of high-risk individuals for screening, diagnosis and treatment; molecular diagnoses of subgroups, and targeted treatment according to molecular subtypes. It is imperative to balance the need of finding the best evidence with that of promoting well-being of patients while keeping in mind the importance of quaternary prevention (Table 2).

**Table 2:** Summary of prevention, management and research strategies for ME/CFS according to stage of disease.

**Stage**	**Predisposition and triggering of disease (Disease onset)**	**Prodromal period and early disease**	**Established disease**
Prevention	Primary: avoidance of further stressors; adequate rest; prioritisation of recovery of initial illness	Secondary: early detection; early intervention allowing recovery time	Tertiary: reduction of long-term morbidity/disability; rehabilitation
Management	Rest; Pacing activities	Early diagnosis; treatment of trigger infection; treatment of immune/CNS/other dysfunction; reduction in symptom severity (using drugs or non-pharmacological interventions); rest; pacing	Interventions aiming to restore homeostatic balance; symptomatic relief Personalised approach Future: treatments for multi-systemic dysfunctions
Research	Risk factor studies: genetic; environmental; life-style studies Cohort studies: following-up population after outbreaks (e.g., SARS-CoV-2), or proxy models (e.g., immune treatment for diseases that induce chronic fatigue)	Large scale longitudinal cohort studies focusing on early disease pathophysiology and interventions Use of electronic health record data Biobank samples and data, existing disease cohorts	Studies on perpetuation of systemic and molecular abnormalities Studies focusing on phenotypic subtypes: using “omics” technologies to aid personalised recovery

## Additional Considerations

### General and Tailored Approaches

The nature of persistent dysfunctions, and whether they can be controlled or resolved, may be central to prognosis and treatment in ME/CFS. For example, in those with more severe post-exertional symptoms (more likely affected by autonomic, neuro-endocrine, and energy metabolism dysfunction), energy management, through pacing and sensible rest, are essential to allow the body to enact its recovery potential, combined with specific treatments to address systems dysfunctions. Those with long illness duration, but with milder symptoms which are improving, may become more tolerant to exertion, feel more energetic and have less cognitive dysfunction or “brain fog”; additionally, their post-exertional symptoms may be less pronounced or be limited to major activities. Such cases could benefit from a program of individually tailored, paced, stepwise and increasing exposure to activities. However, those in whom disease is progressing unfavourably may benefit from medical treatments targeted at specific dysfunctions alongside rehabilitation. As in many chronic debilitating diseases, psychological therapies have a role in supporting individuals through their chronic illness as part of an important supplementary component of holistic medical care that includes a personal approach to management and treatment.

### Severe or Complicated ME/CFS

Approximately 25% of ME/CFS patients will develop a severe form of disease, rendering them house- or bed-bound (76). Difficulty accessing this particular portion of the patient community, in both clinical practise and research, further exacerbates inadequate access to specialist care (106), selection bias and non-generalisability of results (107, 108). Access to medical services is often limited, augmented by the lack of knowledge among healthcare practitioners due to both a lack of appropriate training in medical school and widespread scepticism concerning the disease (109); many healthcare professionals are reluctant to give an ME/CFS diagnosis, especially in the early stages of the disease.

Treatment for severe or complicated ME/CFS is more challenging, as patients may have achieved an advanced state of homeostatic dysregulation with increasing multi-system dysfunction and multi-system complications. The previous state of chronic inflammation may now be subdued, while the body may enter a hypometabolic state (48). This state includes the slowing of physiological pathways and reduction of energy output, with chronic cell and system malfunction.

In addition to the measures used in early and less severe cases, any treatment approach needs to consider specific mechanisms leading to and perpetuating cell dysfunction (e.g., those associated with endothelial dysfunction and cytopathic hypoxia). These treatments may include strategies aimed at reversing neuro-endocrine and metabolic abnormalities, and at rehabilitation. Examples of interventions include neurological rehabilitation (e.g., gentle or passive physiotherapy), nutritional rehabilitation (which might need to involve enteral feeding), and those targeting circulatory dysfunction (e.g., treatment of hypotension and postural tachycardia and other manifestations of orthostatic intolerance), and the various consequences of prolonged illness (e.g., screening for and treatment of osteoporosis). Severely affected patients have restricted activity, often struggling with self-care, and needing support from carers and from a multi-disciplinary health-team. For this sub-group of patients, effective input and support from social, educational and occupational health services may be even more important, alongside a range of rehabilitative interventions.

Treatment approaches targeting specific energy metabolic dysfunctions, as well as specific nutritional and hormonal supplements, may also play a restorative role; however, these still need development and validation before they can be used beyond individually tailored approaches.

### Research at Different Disease Stages

Several research questions still need answers, requiring different strategies and ways of selecting research participants. Researchers should consider the advantages of restricting the study population of cases to those who meet diagnostic criteria with higher specificity (110) and of case stratification, including sub-grouping of cases into disease stages. Alternatively, research could focus on a specific stage. While there is no doubt that molecular research is essential to revealing disease pathways and for biomarker discovery, other types of research, such as clinical, epidemiological, environmental, health service, policy and education are essential for better disease recognition, prevention, diagnosis, and treatment while an emphasis on cross-cultural studies may encourage a more standardised view of ME/CFS internationally.

Refocusing to include pre-clinical or “invisible” stages of illness can be hugely beneficial for the study of disease. One such example is Alzheimer's where, over the past decade, a conceptual shift to consider the disease as a continuum has occurred and, along with the discovery of biomarkers, has re-focused the research agenda towards the pre-clinical stage and early intervention (111). Similarly, we suggest that a re-focus of the ME/CFS research agenda towards the pre-clinical stage (by way of larger prospective cohort studies), may contribute to revealing potential risk factors to support primary prevention efforts.

## Conclusions

The conceptualisation of ME/CFS into disease stages helps to understand disease pathways, their operation, and interconnections along the disease course, and therefore to support the planning of public health and clinical interventions, as well as targeted research.

Discrepancies in the use of diagnostic criteria and sampling methods have led to much variation in research results in ME/CFS and this is mirrored in the care of those affected. As research is directed towards biomedical, systems and molecular investigations, the need for better disease stratification becomes more evident, for both research purposes and clinical practise. It is important, therefore, to consider ME/CFS as a continuum and to examine the different stages patients go through throughout the course of their disease, their severity, and the presence and degree of complications as key parameters for stratification.

Pathophysiological patterns and changes along and across disease stages result in the expression of different, albeit overlapping, phenotypes and any approach to diagnosis, subgrouping, and clinical management will vary according to these phenotypes, as will research questions and the selection of patients for research. Loss of specificity caused by the amalgamation of people with ME/CFS with those with chronic fatigue due to other causes in observational and interventional studies is problematic. Similarly, ignoring different subgroups of ME/CFS, including those related to disease stage, will have an impact on research outputs and their interpretation when investigating disease mechanisms and pathways, including clinical trials.

The concepts of determinants of health and levels of intervention are useful as they provide a framework that can be used to guide disease prevention and management, as well as research direction. The recruitment of individuals for research at the pre-illness stage could be invaluable to understanding the biological mechanisms at play before, during, and after an insult. Longitudinal studies would help to determine where individuals are in terms of the natural course of the disease and to encourage the investigation of abnormalities and of treatments that take into account disease stage, here considered as an additional category for subtyping.

This paper seeks to re-focus research and treatment management efforts. While we wait for detailed mechanisms to be identified, acquired transferrable knowledge, and good health care are required to ensure safe, high quality care for those who are ill.

## Data Availability Statement

Data are available upon request from the authors.

## Author Contributions

LN and EL conceived the paper. SO'B contributed to drafting, referencing, and formatting. All authors contributed to drafting and to revising the manuscript and approved the final version to be published.

## Funding

Research reported in this manuscript was supported by the National Institutes of Health under award number 2R01AI103629.

## Author Disclaimer

The content is solely the responsibility of the authors and does not necessarily represent the official views of the NIH.

## Conflict of Interest

The authors declare that the research was conducted in the absence of any commercial or financial relationships that could be construed as a potential conflict of interest.

## Publisher's Note

All claims expressed in this article are solely those of the authors and do not necessarily represent those of their affiliated organizations, or those of the publisher, the editors and the reviewers. Any product that may be evaluated in this article, or claim that may be made by its manufacturer, is not guaranteed or endorsed by the publisher.

## References

[1] Institute of Medicine (IOM). Beyond Myalgic Encephalomyelitis/Chronic Fatigue Syndrome: Redefining an Illness. Washington, DC: The National Academies Press (2015). 25695122

[2] Lacerda EM Geraghty K Kingdon CC Palla L Nacul L. A logistic regression analysis of risk factors in ME/CFS pathogenesis. BMC Neurol. (2019) 19:275. 10.1186/s12883-019-1468-2 31699051 PMC6839177

[3] Rasa S Nora-Krukle Z Henning N Eliassen E Shikova E Harrer T. Chronic viral infections in myalgic encephalomyelitis/chronic fatigue syndrome (ME/CFS). J Transl Med. (2018) 16:268. 10.1186/s12967-018-1644-y 30285773 PMC6167797

[4] Sepúlveda N Carneiro J Lacerda E Nacul L. Myalgic encephalomyelitis/chronic fatigue syndrome as a hyper-regulated immune system driven by an interplay between regulatory t cells and chronic human herpesvirus infections. Front Immunol. (2019) 10. 10.3389/fimmu.2019.02684 31824487 PMC6883905

[5] Shikova E Reshkova V Kumanova A Raleva S Alexandrova D Capo N. Cytomegalovirus, Epstein-Barr virus, and human herpesvirus-6 infections in patients with myalgic Encephalomyelitis/chronic fatigue syndrome. J Med Virol. (2020) 92:3682–8. 10.1002/jmv.25744 32129496 PMC7687071

[6] Phelan J Grabowska AD Sepúlveda N. A potential antigenic mimicry between viral and human proteins linking Myalgic Encephalomyelitis/Chronic Fatigue Syndrome (ME/CFS) with autoimmunity: the case of HPV immunization. Autoimmun Rev. (2020) 19:102487. 10.1016/j.autrev.2020.102487 32062027

[7] Nacul L Lacerda EM Kingdon CC Curran H Bowman EW. How have selection bias and disease misclassification undermined the validity of myalgic encephalomyelitis/chronic fatigue syndrome studies? J Health Psychol. (2017) 24:1765–9. 10.1177/1359105317695803 28810428 PMC5581258

[8] White PD Goldsmith KA Johnson AL Potts L Walwyn R DeCesare JC. Comparison of adaptive pacing therapy, cognitive behaviour therapy, graded exercise therapy, and specialist medical care for chronic fatigue syndrome (PACE): a randomised trial. Lancet. (2011) 377:823–36. 10.1016/S0140-6736(11)60096-2 21334061 PMC3065633

[9] Fukuda K Straus SE Hickie I Sharpe MC Dobbins JG Komaroff A. The chronic fatigue syndrome: a comprehensive approach to its definition and study. Ann Intern Med. (1994) 121:953–9. 10.7326/0003-4819-121-12-199412150-00009 7978722

[10] Carruthers BM Jain AK DeMeirleir KL Peterson D Klimas NG Lerner AM. Myalgic encephalomyelitis / chronic fatigue syndrome : clinical working case definition, diagnostic and treatment protocols. J Chronic Fatigue Syndr. (2003) 11:7–36. 10.1300/J092v11n01_02

[11] Katz BZ Shiraishi Y Mears CJ Binns HJ Taylor R. Chronic fatigue syndrome following infectious mononucleosis in adolescents: a prospective cohort study. Pediatrics. (2009) 124:189. 10.1542/peds.2008-1879 19564299 PMC2756827

[12] Buchwald DS Rea TD Katon WJ Russo JE Ashley RL. Acute infectious mononucleosis: characteristics of patients who report failure to recover. Am J Med. (2000) 109:531–7. 10.1016/S0002-9343(00)00560-X 11063953

[13] Hickie I Davenport T Wakefield D Vollmer-Conna U Cameron B Vernon SD. Post-infective and chronic fatigue syndromes precipitated by viral and non-viral pathogens: prospective cohort study. BMJ. (2006) 333:575. 10.1136/bmj.38933.585764.AE 16950834 PMC1569956

[14] Nacul L O'Boyle S Palla L Nacul FE Mudie K Kingdon CC. How Myalgic Encephalomyelitis/Chronic Fatigue Syndrome (ME/CFS) Progresses: the natural history of ME/CFS. Front Neurol. (2020) 11:826. 10.3389/fneur.2020.00826 32849252 PMC7431524

[15] Janal MN Ciccone DS Natelson BH. Sub-typing CFS patients on the basis of minor symptoms. Biol Psychol. (2006) 73:124–31. 10.1016/j.biopsycho.2006.01.003 16473456

[16] Reynolds GK Lewis DP Richardson AM Lidbury BA. Comorbidity of postural orthostatic tachycardia syndrome and chronic fatigue syndrome in an Australian cohort. J Intern Med. (2014) 275:409–17. 10.1111/joim.12161 24206536

[17] Zhang L Gough J Christmas D Mattey DL Richards SC Main J. Microbial infections in eight genomic subtypes of chronic fatigue syndrome/myalgic encephalomyelitis. J Clin Pathol. (2010) 63:156–64. 10.1136/jcp.2009.072561 19955554 PMC2921262

[18] Kerr JR Burke B Petty R Gough J Fear D Mattey DL. Seven genomic subtypes of chronic fatigue syndrome/myalgic encephalomyelitis: a detailed analysis of gene networks and clinical phenotypes. J Clin Pathol. (2008) 61:730–9. 10.1136/jcp.2007.053553 18057078

[19] Nagy-Szakal D Barupal DK Lee B Che X Williams BL Kahn EJR. Insights into myalgic encephalomyelitis/chronic fatigue syndrome phenotypes through comprehensive metabolomics. Sci Rep. (2018) 8:10056. 10.1038/s41598-018-28477-9 29968805 PMC6030047

[20] Santamarina-Perez P Eiroa-Orosa FJ Freniche V Moreno-Mayos A Alegre J Saez N. Length of illness does not predict cognitive dysfunction in chronic fatigue syndrome. Appl Neuropsychol. (2011) 18:216–22. 10.1080/09084282.2011.595448 21846221

[21] Bakken IJ Tveito K Gunnes N Ghaderi S Stoltenberg C Trogstad L. Two age peaks in the incidence of chronic fatigue syndrome/myalgic encephalomyelitis: a population-based registry study from Norway 2008-2012. BMC Med. (2014) 12:167. 10.1186/PREACCEPT-1843684980131041 25274261 PMC4189623

[22] Prins JB Van Der Meer JWM Bleijenberg G. Chronic fatigue syndrome. Lancet. (2006) 367:346–55. 10.1016/S0140-6736(06)68073-2 16443043

[23] Nacul LC Lacerda EM Pheby D Campion P Molokhia M Fayyaz S. Prevalence of myalgic encephalomyelitis/chronic fatigue syndrome (ME/CFS) in three regions of England: a repeated cross-sectional study in primary care. BMC Med. (2011) 9:91. 10.1186/1741-7015-9-91 21794183 PMC3170215

[24] Monro JA Puri BK. A Molecular neurobiological approach to understanding the aetiology of chronic fatigue syndrome (myalgic encephalomyelitis or systemic exertion intolerance disease) with treatment implications. Mol Neurobiol. (2018) 55:7377–88. 10.1007/s12035-018-0928-9 29411266 PMC6096969

[25] Naess H Nyland M Hausken T Follestad I Nyland HI. Chronic fatigue syndrome after Giardia enteritis: clinical characteristics, disability and long-term sickness absence. BMC Gastroenterol. (2012) 12:13. 10.1186/1471-230X-12-13 22316329 PMC3292445

[26] Levine PH Jacobson S Pocinki AG Cheney P Peterson D Connelly RR. Clinical, epidemiologic, and virologic studies in four clusters of the chronic fatigue syndrome. Arch Intern Med. (1992) 152:1611–6. 10.1001/archinte.152.8.1611 1323246

[27] Levine PH Snow PG Ranum BA Paul C Holmes MJ. Epidemic neuromyasthenia and chronic fatigue syndrome in West Otago, New Zealand. Arch Intern Med. (1997) 157:750. 10.1001/archinte.1997.00440280064005 9125006

[28] Underhill RA. Myalgic encephalomyelitis, chronic fatigue syndrome: an infectious disease. Med Hypotheses. (2015) 85:765–73. 10.1016/j.mehy.2015.10.011 26604026

[29] Gow JW Hagan S Herzyk P Cannon C Behan PO Chaudhuri A. A gene signature for post-infectious chronic fatigue syndrome. BMC Med Genomics. (2009) 2:38. 10.1186/1755-8794-2-38 19555476 PMC2716361

[30] Kerr JR Petty R Burke B Gough J Fear D Sinclair LI. Gene expression subtypes in patients with chronic fatigue syndrome/myalgic encephalomyelitis. J Infect Dis. (2008) 197:1171–84. 10.1086/533453 18462164

[31] Chu L Valencia IJ Garvert DW Montoya JG. Onset patterns and course of myalgic encephalomyelitis/chronic fatigue syndrome. Front Pediatr. (2019) 7:12. 10.3389/fped.2019.00012 30805319 PMC6370741

[32] Heim C Nater UM Maloney E Boneva R Jones JF Reeves WC. Childhood trauma and risk for chronic fatigue syndrome: association with neuroendocrine dysfunction. Arch Gen Psychiatry. (2009) 66:72–80. 10.1001/archgenpsychiatry.2008.508 19124690

[33] Nater UM Maloney E Heim C Reeves WC. Cumulative life stress in chronic fatigue syndrome. Psychiatry Res. (2011) 189:318–20. 10.1016/j.psychres.2011.07.015 21840607

[34] Harvey SB Wadsworth M Wessely S Hotopf M. The relationship between prior psychiatric disorder and chronic fatigue: evidence from a national birth cohort study. Psychol Med. (2008) 38:933. 10.1017/S0033291707001900 17976252 PMC3196526

[35] Shepherd C Chaudhuri A. ME/CFS/PVFS: An Exploration of the Key Clinical Issues. 11th ed. Gawco: The ME Association (2019). p. 152.

[36] CDC. Long-Term Effects of COVID-19 (2020).

[37] Marshall M. The lasting misery of coronavirus long-haulers. Nature. (2020) 585:339–41. 10.1038/d41586-020-02598-6 32929257

[38] Yelin D Wirtheim E Vetter P Kalil AC Bruchfeld J Runold M. Long-term consequences of COVID-19: research needs. Lancet. (2020) 20:1115–17. 10.1016/S1473-3099(20)30701-5 32888409 PMC7462626

[39] Black PH. Immune system-central nervous system interactions: effect and immunomodulatory consequences of immune system mediators on the brain. Antimicrob Agents Chemother. (1994) 38:7–12. 10.1128/AAC.38.1.7 8141583 PMC284389

[40] Prüss H Tedeschi A Thiriot A Lynch L Loughhead SM Stutte S. Spinal cord injury-induced immunodeficiency is mediated by a sympathetic-neuroendocrine adrenal reflex. Nat Neurosci. (2017) 20:1549–59. 10.1038/nn.4643 28920935

[41] Wrona D. Neural–immune interactions: An integrative view of the bidirectional relationship between the brain and immune systems. J Neuroimmunol. (2006) 172:38–58. 10.1016/j.jneuroim.2005.10.017 16375977

[42] Natelson BH Weaver SA Tseng CL Ottenweller JE. Spinal fluid abnormalities in patients with chronic fatigue syndrome. Clin Diagn Lab Immunol. (2005) 12:52–5. 10.1128/CDLI.12.1.52-55.2005 15642984 PMC540195

[43] Schutzer SE Angel TE Liu T Schepmoes AA Clauss TR Adkins JN. Distinct cerebrospinal fluid proteomes differentiate post-treatment lyme disease from chronic fatigue syndrome. PLoS ONE. (2011) 6:e17287. 10.1371/journal.pone.0017287 21383843 PMC3044169

[44] Chaudhuri A Behan PO. Fatigue and basal ganglia. J Neurol Sci. (2000) 179:34–42. 10.1016/S0022-510X(00)00411-1 11054483

[45] Bested AC Saunders PR Logan AC. Chronic fatigue syndrome: neurological findings may be related to blood–brain barrier permeability. Med Hypotheses. (2001) 57:231–7. 10.1054/mehy.2001.1306 11461179

[46] Klimas NG Broderick G Fletcher MA. Biomarkers for chronic fatigue. Brain Behav Immun. (2012) 26:1202-10. 10.1016/j.bbi.2012.06.006 22732129 PMC5373648

[47] Komaroff AL. Advances in understanding the pathophysiology of chronic fatigue syndrome. JAMA. (2019) 322:499–500. 10.1001/jama.2019.8312 31276153

[48] Naviaux RK Naviaux JC Li K Bright AT Alaynick WA Wang L. Metabolic features of chronic fatigue syndrome. Proc Natl Acad Sci USA. (2016) 113:5472–80. 10.1073/pnas.1607571113 27573827 PMC5027464

[49] Kennedy G Spence VA McLaren M Hill A Underwood C Belch JJ. Oxidative stress levels are raised in chronic fatigue syndrome and are associated with clinical symptoms. Free Radic Biol Med. (2005) 39:584–9. 10.1016/j.freeradbiomed.2005.04.020 16085177

[50] Fluge Ø Mella O Bruland O Risa K Dyrstad SE Alme K. Metabolic profiling indicates impaired pyruvate dehydrogenase function in myalgic encephalopathy/chronic fatigue syndrome. JCI Insight. (2016) 1:e89376. 10.1172/jci.insight.89376 28018972 PMC5161229

[51] Balinas C Cabanas H Staines D Marshall-Gradisnik S. Transient receptor potential melastatin 2 channels are overexpressed in myalgic encephalomyelitis/chronic fatigue syndrome patients. J Transl Med. (2019) 17:401. 10.1186/s12967-019-02155-4 31796045 PMC6891975

[52] van Campen CM Rowe PC Visser FC. Reductions in cerebral blood flow can be provoked by sitting in severe myalgic encephalomyelitis/chronic fatigue syndrome patients. Healthcare. (2020) 8:394. 10.3390/healthcare8040394 33050553 PMC7712289

[53] Beaumont A Burton AR Lemon J Bennett BK Lloyd A Vollmer-Conna U. Reduced cardiac vagal modulation impacts on cognitive performance in chronic fatigue syndrome. PLoS ONE. (2012) 7:e49518. 10.1371/journal.pone.0049518 23166694 PMC3498107

[54] Cambras T Castro-Marrero JJ Cleofé Zaragoza M Díez-Noguera A Alegre JJ Zaragoza MC. Circadian rhythm abnormalities and autonomic dysfunction in patients with Chronic Fatigue Syndrome/Myalgic Encephalomyelitis. PLoS ONE. (2018) 13:e0198106. 10.1371/journal.pone.0198106 29874259 PMC5991397

[55] Mueller C Lin JC Sheriff S Maudsley AA Younger JW. Evidence of widespread metabolite abnormalities in Myalgic encephalomyelitis/chronic fatigue syndrome: assessment with whole-brain magnetic resonance spectroscopy. Brain Imaging Behav. (2019) 14:562–72. 10.1007/s11682-018-0029-4 30617782 PMC6612467

[56] Rowe PC Fontaine KR Lauver M Jasion SE Marden CL Moni M. Neuromuscular strain increases symptom intensity in chronic fatigue syndrome. PLoS ONE. (2016) 11:e0159386. 10.1371/journal.pone.0159386 27428358 PMC4948885

[57] Glassford JAG. The neuroinflammatory etiopathology of Myalgic Encephalomyelitis/Chronic Fatigue Syndrome (ME/CFS). Front Physiol. (2017) 8:1–9. 10.3389/fphys.2017.00088 28261110 PMC5314655

[58] Rowe PC Fontaine KR Violand RL. Neuromuscular strain as a contributor to cognitive and other symptoms in chronic fatigue syndrome: hypothesis and conceptual model. Front Physiol. (2013) 4:115. 10.3389/fphys.2013.00115 23720638 PMC3655286

[59] Dubin AE Patapoutian A. Nociceptors: the sensors of the pain pathway. J Clin Invest. (2010) 120:3760–72. 10.1172/JCI42843 21041958 PMC2964977

[60] Morris G Berk M Puri BK. A comparison of neuroimaging abnormalities in multiple sclerosis, major depression and chronic fatigue syndrome (myalgic encephalomyelitis): is there a common cause? Mol Neurobiol. (2018) 55:3592–609. 10.1007/s12035-017-0598-z 28516431 PMC5842501

[61] Albrecht DS Forsberg A Sandström A Bergan C Kadetoff D Protsenko E. Brain glial activation in fibromyalgia – a multi-site positron emission tomography investigation. Brain Behav Immun. (2019) 75:72–83. 10.1016/j.bbi.2018.09.018 30223011 PMC6541932

[62] Hornig M Montoya JG Klimas NG Levine S Felsenstein D Bateman L. Distinct plasma immune signatures in ME/CFS are present early in the course of illness. Sci Adv. (2015) 1:1–10. 10.1126/sciadv.1400121 26079000 PMC4465185

[63] Nisenbaum R Jones JF Unger ER Reyes M Reeves WC. A population-based study of the clinical course of chronic fatigue syndrome. Heal Qual Life Outcomes. (2003) 1:49. 10.1186/1477-7525-1-49 14613572 PMC269990

[64] Russell L Broderick G Taylor R Fernandes H Harvey J Barnes Z. Illness progression in chronic fatigue syndrome: a shifting immune baseline. BMC Immunol. (2016) 17:3. 10.1186/s12865-016-0142-3 26965484 PMC4785654

[65] Nacul LC Lacerda EM Sakellariou D. Is there an association between exposure to chemicals and chronic fatigue syndrome? Review of the evidence. Bull IACFS/ME. (2009) 17:3–15. Available online at: https://drive.google.com/drive/folders/1hR9sz4qzq5szOWzEDEX8zwAmbyo2gTJ1

[66] Dahlgren G Whitehead M. Policies and strategies to promote social equity in health Background document to WHO – strategy paper for Europe, No 2007:14. In: Strategy Paper for Europe. Stockholm: Arbetsrapport, Institute for Futures Studies (1991).

[67] Aaron LA Herrell R Ashton S Belcourt M Schmaling K Goldberg J. Comorbid clinical conditions in chronic fatigue. A co-twin control study. J Gen Intern Med. (2001) 16:24–31. 10.1111/j.1525-1497.2001.03419.x 11251747 PMC1495162

[68] Buchwald D Herrell R Ashton S Belcourt M Schmaling K Sullivan P. A twin study of chronic fatigue. Psychosom Med. (2001) 63:936–43. 10.1097/00006842-200111000-00012 11719632

[69] Albright F Light K Light A Bateman L Cannon-Albright LA. Evidence for a heritable predisposition to Chronic Fatigue Syndrome. BMC Neurol. (2011) 11:62. 10.1186/1471-2377-11-62 21619629 PMC3128000

[70] Underhill RA O'Gorman R. Prevalence of chronic fatigue syndrome and chronic fatigue within families of CFS patients. J Chronic Fatigue Syndr. (2006) 13:3–13. 10.1300/J092v13n01_02

[71] Russell A Hepgul N Nikkheslat N Borsini A Zajkowska Z Moll N. Persistent fatigue induced by interferon-alpha: a novel, inflammation-based, proxy model of chronic fatigue syndrome. Psychoneuroendocrinology. (2019) 100:276–85. 10.1016/j.psyneuen.2018.11.032 30567628 PMC6350004

[72] Sudre CH Murray B Varsavsky T Graham MS Penfold RS Bowyer RC. Attributes and predictors of long COVID. Nat Med. (2021) 72:626–31. 10.1038/s41591-021-01361-2 33692530 PMC7611399

[73] Goërtz YMJ Van Herck M Delbressine JM Vaes AW Meys R Machado FVC. Persistent symptoms 3 months after a SARS-CoV-2 infection: the post-COVID-19 syndrome? ERJ Open Res. (2020) 6:00542-2020. 10.1183/23120541.00542-2020 33257910 PMC7491255

[74] Last J. A Dictionary of Epidemiology. New York, NY: Oxford University Press (2014) p. 376.

[75] Rowe PC Underhill RA Friedman KJ Gurwitt A Medow MS Schwartz MS. Myalgic encephalomyelitis/ chronic fatigue syndrome diagnosis and management in young people: a primer. Front Pediatr. (2017) 5:121. 10.3389/fped.2017.00121 28674681 PMC5474682

[76] National Institute of Clinical Excellence. Chronic Fatigue Syndrome/Myalgic Encephalomyelitis (or Encephalopathy): Diagnosis and Management of CFS/ME in Adults and Children. NICE Guidelines, London (2014).

[77] Natelson BH. Your Symptoms Are Real. Hoboken, NJ: John Wiley & Sons, Inc. (2008) p. 280.

[78] Goudsmit EM Nijs J Jason LA Wallman KE. Pacing as a strategy to improve energy management in myalgic encephalomyelitis/chronic fatigue syndrome: a consensus document. Disabil Rehabil. (2012) 34:1140–7. 10.3109/09638288.2011.635746 22181560

[79] Jason L Muldowney K Torres-Harding S. The Energy Envelope Theory and myalgic encephalomyelitis/chronic fatigue syndrome. AAOHN J. (2008) 56:189–95. 10.1177/216507990805600502 18578185

[80] Higginbottom K. The Price of Presenteeism. Forbes (2018). Available online at: https://www.forbes.com/sites/karenhigginbottom/2018/04/20/the-price-of-presenteeism-2/#19a8d6e17f9c

[81] Stewart WF Ricci JA Chee E Morganstein D Lipton R. Lost productive time and cost due to common pain conditions in the US workforce. JAMA. (2003) 290:2443. 10.1001/jama.290.18.2443 14612481

[82] Carruthers BM van de Sande MI Klimas NG Broderick G Bell DS Carlo-Stella N. Myalgic Encephalomyelitis - Adult & Paediatrics - International Consensus for Medical Practitioners. (2012). Available online at: http://hetalternatief.org/ICC%20primer%202012.pdf

[83] Nacul L Authier FJ Scheibenbogen C Lorusso L Helland IB Martin JA. European Network on Myalgic Encephalomyelitis/Chronic Fatigue Syndrome (EUROMENE): expert consensus on the diagnosis, service provision, and care of people with ME/CFS in Europe. Medicina. (2021) 57:510. 10.3390/medicina57050510 34069603 PMC8161074

[84] CDC. Treatment of ME/CFS (2021).

[85] Shepherd, C,. Management. The ME Association. Available online at: https://meassociation.org.uk/about-what-is-mecfs/management/

[86] US ME/CFS Clinician Coalition. Diagnosing and Treating Myalgic Encephalomyelitis/ Chronic Fatigue Syndrome (ME/CFS) (2020).

[87] Office for National Statistics. The Prevalence of Long COVID Symptoms and COVID-19 Complications (2020).

[88] Gorna R MacDermott N Rayner C O'Hara M Evans S Agyen L. Long COVID guidelines need to reflect lived experience. Lancet. (2020) 397:455–7. 10.1016/S0140-6736(20)32705-7 33357467 PMC7755576

[89] Shepherd C. Covid-19, ME/CFS & Post-Covid Syndromes. The ME Association (2020).

[90] The Lancet Neurology. Long COVID: understanding the neurological effects. Lancet Neurol. (2021) 20:247. 10.1016/S1474-4422(21)00059-4 33743226 PMC7969137

[91] NHS England. Your COVID Recovery | Managing Daily Activities (2020).

[92] Torjesen I. NICE advises against using graded exercise therapy for patients recovering from covid-19. BMJ. (2020) 370:m2912. 10.1136/bmj.m2912 32694164

[93] Davis HE Assaf GS McCorkell L Wei H Low RJ Re'em Y. Characterizing long COVID in an international cohort: 7 months of symptoms and their impact. E Clinical Medicine. (2021) 38:101019. 10.1016/j.eclinm.2021.101019 34308300 PMC8280690

[94] Lokugamage A Taylor S Rayner C. Patients' experiences of longcovid are missing from the NHS narrative. BMJ. (2020). Available online at: https://blogs.bmj.com/bmj/2020/07/10/patients-experiences-of-longcovid-are-missing-from-the-nhs-narrative/

[95] Alwan NA Attree E Blair JM Bogaert D Bowen MA Boyle J. From doctors as patients: a manifesto for tackling persisting symptoms of covid-19. BMJ. (2020) 370:33. 10.1136/bmj.m3565 32933949

[96] Missailidis D Sanislav O Allan CY Annesley SJ Fisher PR. Cell-based blood biomarkers for myalgic encephalomyelitis/chronic fatigue syndrome. Int J Mol Sci. (2020) 21:1142. 10.3390/ijms21031142 32046336 PMC7037777

[97] Esfandyarpour R Kashi A Nemat-Gorgani M Wilhelmy J Davis RW. A nanoelectronics-blood-based diagnostic biomarker for myalgic encephalomyelitis/chronic fatigue syndrome (ME/CFS). Proc Natl Acad Sci USA. (2019) 116:10250–7. 10.1073/pnas.1901274116 31036648 PMC6535016

[98] Lacerda EM Bowman EW Cliff JM Kingdon CC King EC Lee J-S. The UK ME/CFS Biobank for biomedical research on Myalgic Encephalomyelitis/Chronic Fatigue Syndrome (ME/CFS) and Multiple Sclerosis. Open J Bioresour. (2017) 4:4. 10.5334/ojb.28 28649428 PMC5482226

[99] De Stavola BL Nitsch D dos Santos Silva I McCormack V Hardy R Mann V. Statistical issues in life course epidemiology. Am J Epidemiol. (2006) 163:84–96. 10.1093/aje/kwj003 16306313

[100] Jamoulle M. Quaternary prevention, an answer of family doctors to overmedicalization. Int J Heal Policy Manag. (2015) 4:61–4. 10.15171/ijhpm.2015.24 25674569 PMC4322627

[101] Martins C Godycki-Cwirko M Heleno B Brodersen J. Quaternary prevention: reviewing the concept. Eur J Gen Pract. (2018) 24:106–11. 10.1080/13814788.2017.1422177 29384397 PMC5795741

[102] Alraek T Lee MS Choi T-Y Cao H Liu J. Complementary and alternative medicine for patients with chronic fatigue syndrome: a systematic review. BMC Complement Altern Med. (2011) 11:87. 10.1186/1472-6882-11-87 21982120 PMC3201900

[103] Marks DF. Special issue on the PACE Trial. J Health Psychol. (2017) 22:1103–5. 10.1177/1359105317722370 28805511

[104] Bell DS. Cellular Hypoxia and Neuro-Immune Fatigue. 1st ed. Livermore, CA: WingSpan Press (2007).

[105] Haendel MA Chute CG Robinson PN. Classification, ontology, and precision medicine. N Engl J Med. (2018) 379:1452–62. 10.1056/NEJMra1615014 30304648 PMC6503847

[106] Mcdermott C Al Haddabi A Akagi H Selby M Cox D Lewith G. What is the current NHS service provision for patients severely affected by chronic fatigue syndrome/myalgic encephalomyelitis? A national scoping exercise. BMJ Open. (2014) 4:5083. 10.1136/bmjopen-2014-005083 24984956 PMC4078780

[107] Pendergrast T Brown A Sunnquist M Jantke R Newton JL Strand EB. Housebound versus nonhousebound patients with myalgic encephalomyelitis and chronic fatigue syndrome. Chronic Illn. (2016) 12:292–307. 10.1177/1742395316644770 27127189 PMC5464362

[108] Strassheim V Lambson RL Hackett KL Newton JL. What is known about severe and very severe chronic fatigue syndrome? A scoping review. Fatigue Biomed Heal Behav. (2017) 5:167–83. 10.1080/21641846.2017.1333185

[109] Bayliss K Goodall M Chisholm A Fordham B Chew-Graham C Riste L. Overcoming the barriers to the diagnosis and management of chronic fatigue syndrome/ME in primary care: a meta synthesis of qualitative studies. BMC Fam Pract. (2014) 15:44. 10.1186/1471-2296-15-44 24606913 PMC3973969

[110] Nacul L Kingdon CC Bowman EW Curran H Lacerda EM Nacul CC Bowman. Differing case definitions point to the need for an accurate diagnosis of myalgic encephalomyelitis/chronic fatigue syndrome. Fatigue Biomed Heal Behav. (2017) 5:1–4. 10.1080/21641846.2017.1273863 29250461 PMC5730342

[111] Dubois B Hampel H Feldman HH Scheltens P Aisen P Andrieu S. Preclinical Alzheimer's disease: definition, natural history, and diagnostic criteria. Alzheimer's Dement. (2016) 12:292–323. 10.1016/j.jalz.2016.02.002 27012484 PMC6417794

